# Supplementary figures and images for: Inhibiting influenza virus transmission using a broadly acting neuraminidase that targets host sialic acids in the upper respiratory tract
Source: mBio. 2024 Jan 11;15(2):e02203-23. doi: 10.1128/mbio.02203-23 (PMC10865980; doi:10.1128/mbio.02203-23)

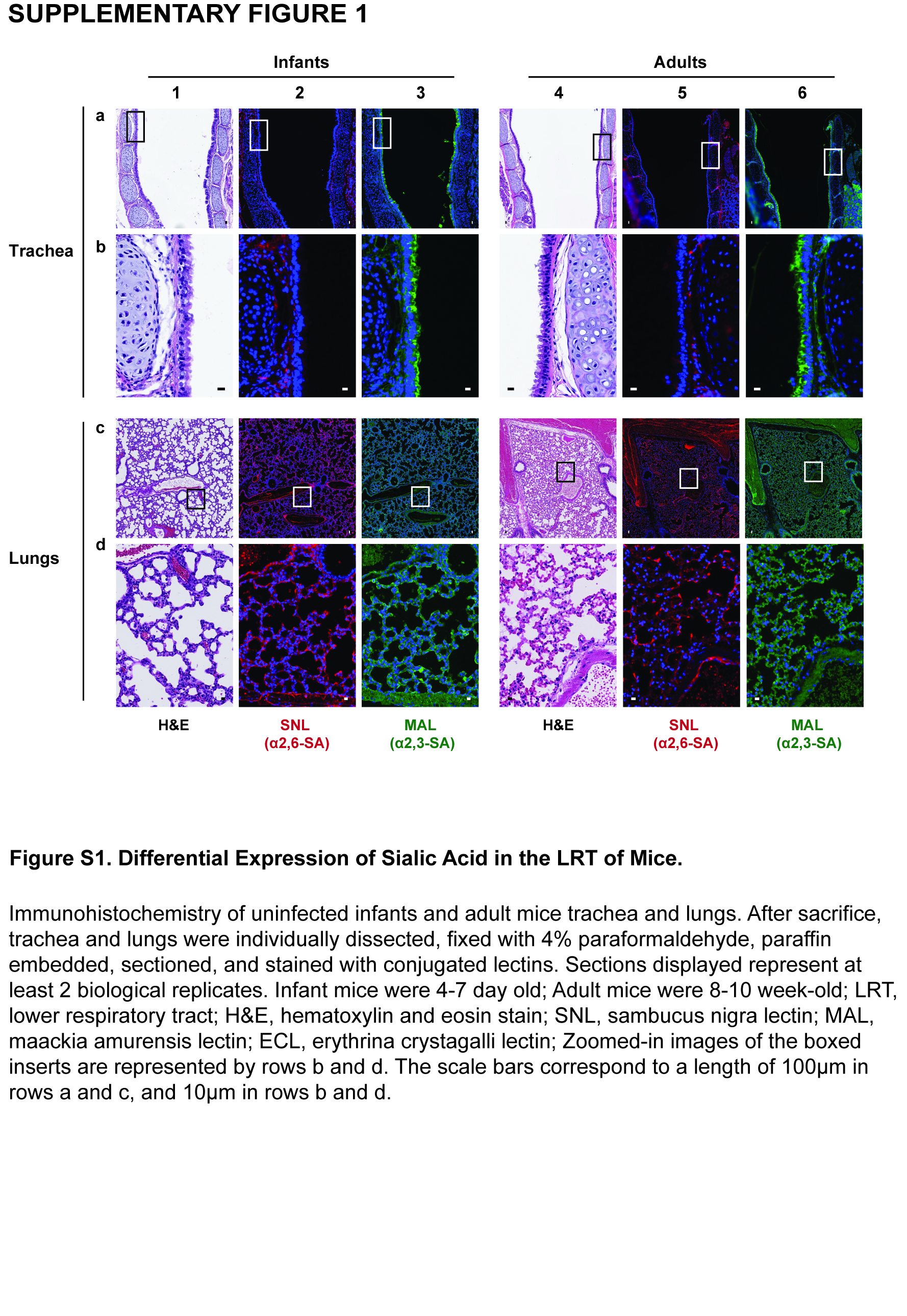

Supplement: Fig. S1 — st6gal1-KO mice. [file mbio.02203-23-s0001.tif]

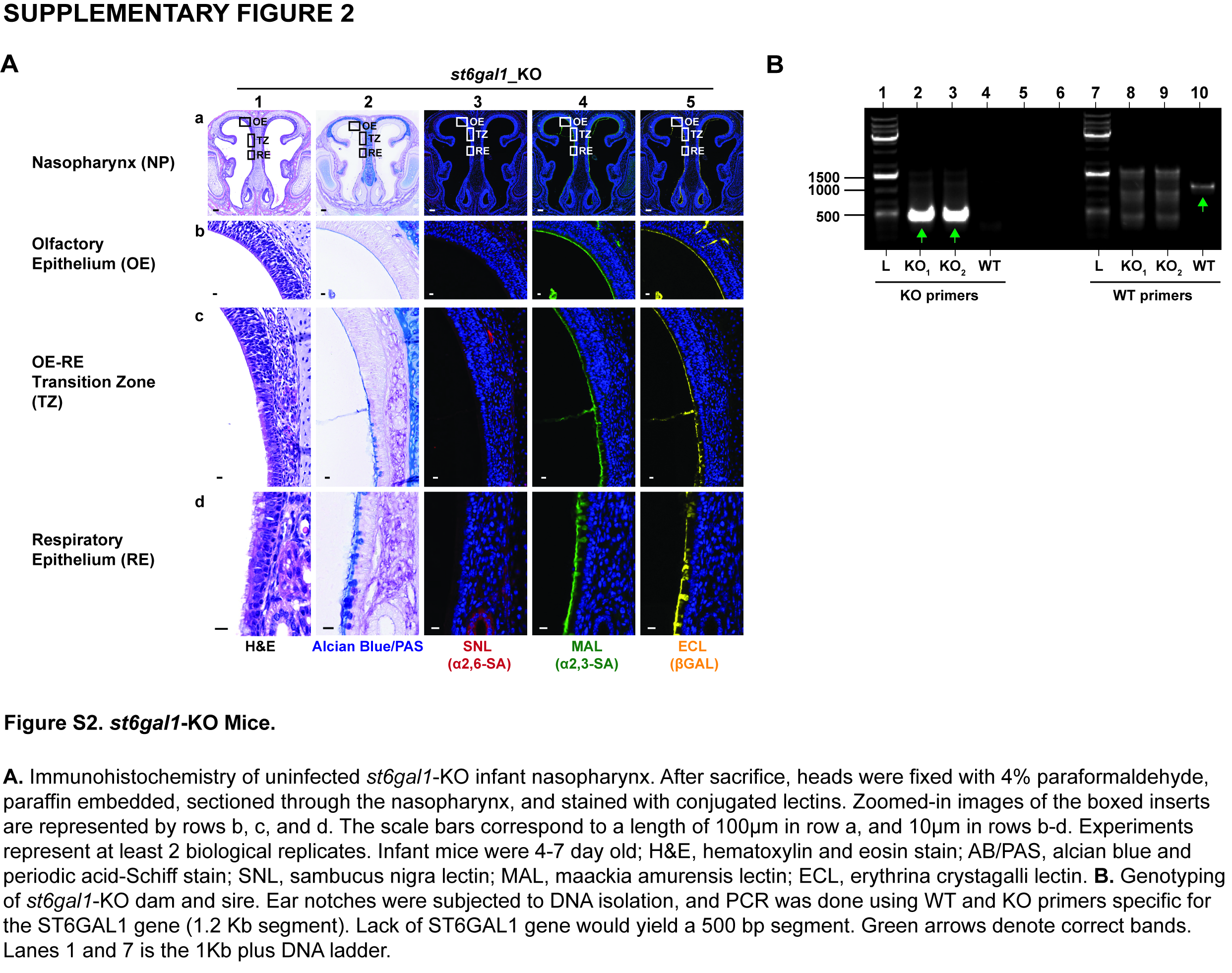

Supplement: Fig. S2 — Differential expression of sialic acid in the LRT of mice. [file mbio.02203-23-s0002.tif]
